# Characterization of the complete mitochondrial genome of the *Hypolimnas misippus* Linnaeus 1764 (Lepidoptera: nymphalidae)

**DOI:** 10.1080/23802359.2023.2246673

**Published:** 2023-08-24

**Authors:** Hong Yu, Fen Wang, Jin Xu

**Affiliations:** Yunnan Academy of Biodiversity, Southwest Forestry University, Kunming, P.R. China

**Keywords:** *Hypolimnas misippus*, mitochondrial genome, phylogenetic analysis

## Abstract

In this study, we sequenced the complete mitochondrial genome (mitogenome) of *Hypolimnas misippus* Linnaeus 1764, which is 15,283 bp in length, containing 13 protein-coding genes (PCGs), 22 transfer RNA genes (tRNAs), two ribosomal RNA genes (rRNAs), and an adenine (A) + thymine (T)-rich (D-loop) region. The overall GC level is 19.8%. The phylogenetic position of *H. misippus* was evaluated using 48 previously published complete mitogenomes, and the results reveals that *H. misippus* is most closely related to *H.bolina*.

## 1. Introduction

The subfamily Nymphalinae (Lepidoptera: Nymphalidae) comprises about 500 species distributed around the world. Several species in the group have been used as model organisms in ecological and evolutionary studies (Brower et al. 2000; Boggs et al. 2003; Kemp et al. 2005). Despite the relatively rich amounts of basic studies about this butterfly group, there is no complete mitochondrial genomes publicly available for *Hypolimnas misippus* Linnaeus. 1764. Therefore, we sequenced the complete mitochondrial DNA genome of *H. misippus* to provide baseline data for this species and also to better understand its relationship within the subfamily Nymphalinae.

## 2. Materials and methods

### 2.1. Sample collection and preservation

**Figure 1.**
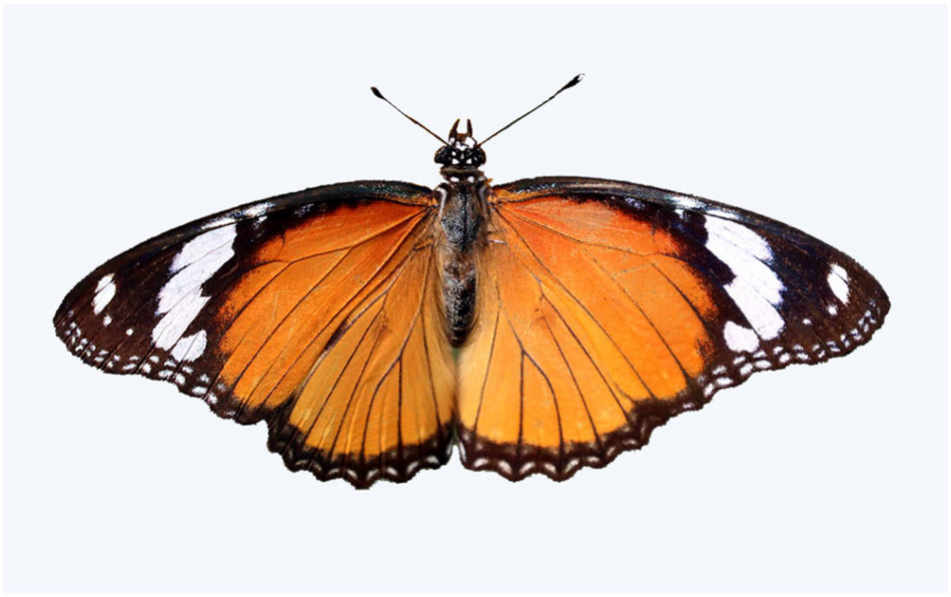
Morphological characteristics of *Hypolimnas misippus* (photographs by JinXu).

Specimens of female *H. misippus* (Figure 1) were collected with hand nets from South China Botanical Garden (23°18′35″N, 113°36'56″E), Guangzhou, China. The wings of female butterflies are orange-yellow, and with a small white spot at the top corner. Two rows of small white spots in pairs are arranged on the outer edge of both wings. The color of the back side of the wings is pale, and there are 3 white spots above the middle chamber. There is one black spot on the middle of the anterior margin of the hindwing and one outside the end of the middle wing vein (Chou 1998).

### 2.2. DNA extraction, sequencing, and assembly

Total genomic DNA was extracted from the thorax muscle of a single individual butterfly using the Sangon Animal genome DNA Extraction Kit (Shanghai, China). The voucher specimen was deposited at Southwest Forestry University under the voucher number SCBGJBJD0916 (Jin Xu, xujin2798@163.com). After DNA isolation, 1 μg of purified DNA was fragmented and used to construct short-insert libraries (insert size ∼350 bp) according to the manufacturer’s instructions (BIG-500). Then DNA libraries were sequenced by Guangzhou BIO&DATA Biotechnologies Inc. (Guangzhou, China) on the BGI-500 Sequencing System (BGI, Shenzhen, China) using PE 150 bp reads. The filtered sequences were assembled using the SPAdes assembler 3.10.0 (Bankevich et al.^2012^). The coverage depth (600 × ∼ 4060 ×, mean: 2709.9 ×, Figure S1) was calculated by Geneious R11 (Kearse et al. 2012) by mapping the total clean reads to de novo assembled plastome.

### 2.3. Annotation and analysis

The mito-genome was annotated using MITOS2(Donath et al.^2019^), BLAST+ (Camacho et al. 2009) and tRNAscan (Schattner et al. 2005). To investigate its taxonomic relationships, a maximum likelihood (ML) phylogeny was reconstructed based on the whole mitogenome of 46 Nymphalinae butterflies and two outgroup butterflies (*Neptis alwina* and *Parthenos sylvia*). All sequences were aligned using the program MAFFT version 7.471 (Katoh and Standley 2013), and then a maximum-likelihood (ML) phylogenetic tree was constructed using FastTree version 2.1.10 with Generalized Time-Reversible (GTR) model, statistical support for branches was tested by Shimodaira-Hasegawa (SH) test (Price 2010).

## 3. Results and discussion

### 3.1. Genomic characterization

**Figure 2.**
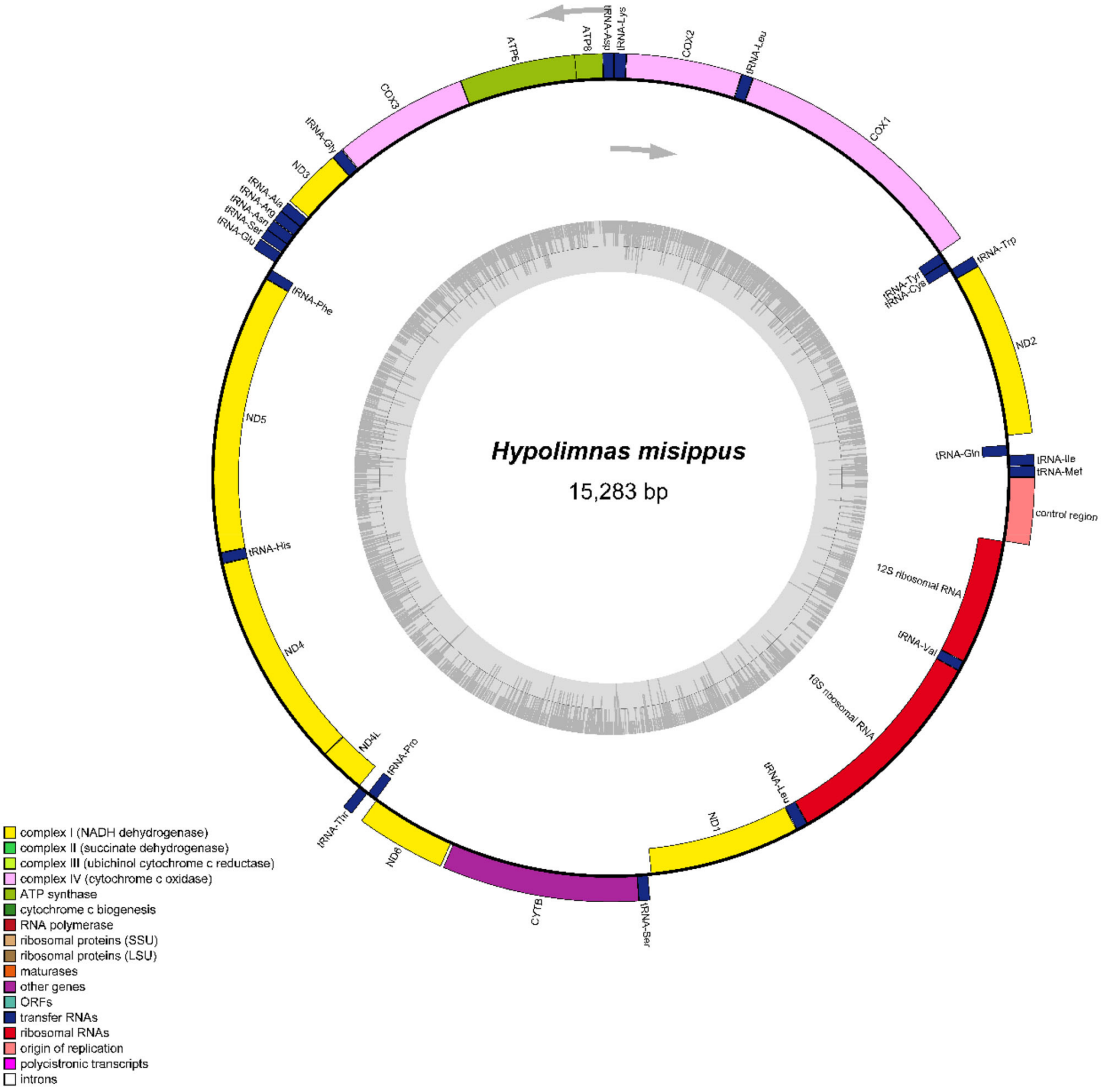
Mitogenome pattern map of *Hypolimnas misippus.*

The circular mitogenome (Genbank acc. no. OL711673) of *H. misippus* (Figure 2) is 15,283 bp in length and contains 13 protein-coding genes (PCGs), 22 transfer RNA genes (tRNAs), two ribosomal RNA genes (rRNAs), and an adenine (A) + thymine (T)-rich (D-loop) region with 91.0% AT content. Among these, 14 genes are encoded on the L-strand, including four PCGs (ND1, ND4, ND4Land ND5), two rRNA genes, eight tRNA genes (tRNA-Cys, tRNA-Gln, tRNA-His, tRNA-Leu, Trna-Phe, tRNAPro, tRNATyr, and tRNAVal). The remaining 23 genes are encoded on the H strand. The gene composition, order, and direction are similar to the mitogenomes of species from same subfamily of Nymphalidae (Shi et al. 2015). The overall AT content (80.2%) is significantly higher than that of GC content (19.8%) in the whole mitogenome.

**Figure 3.**
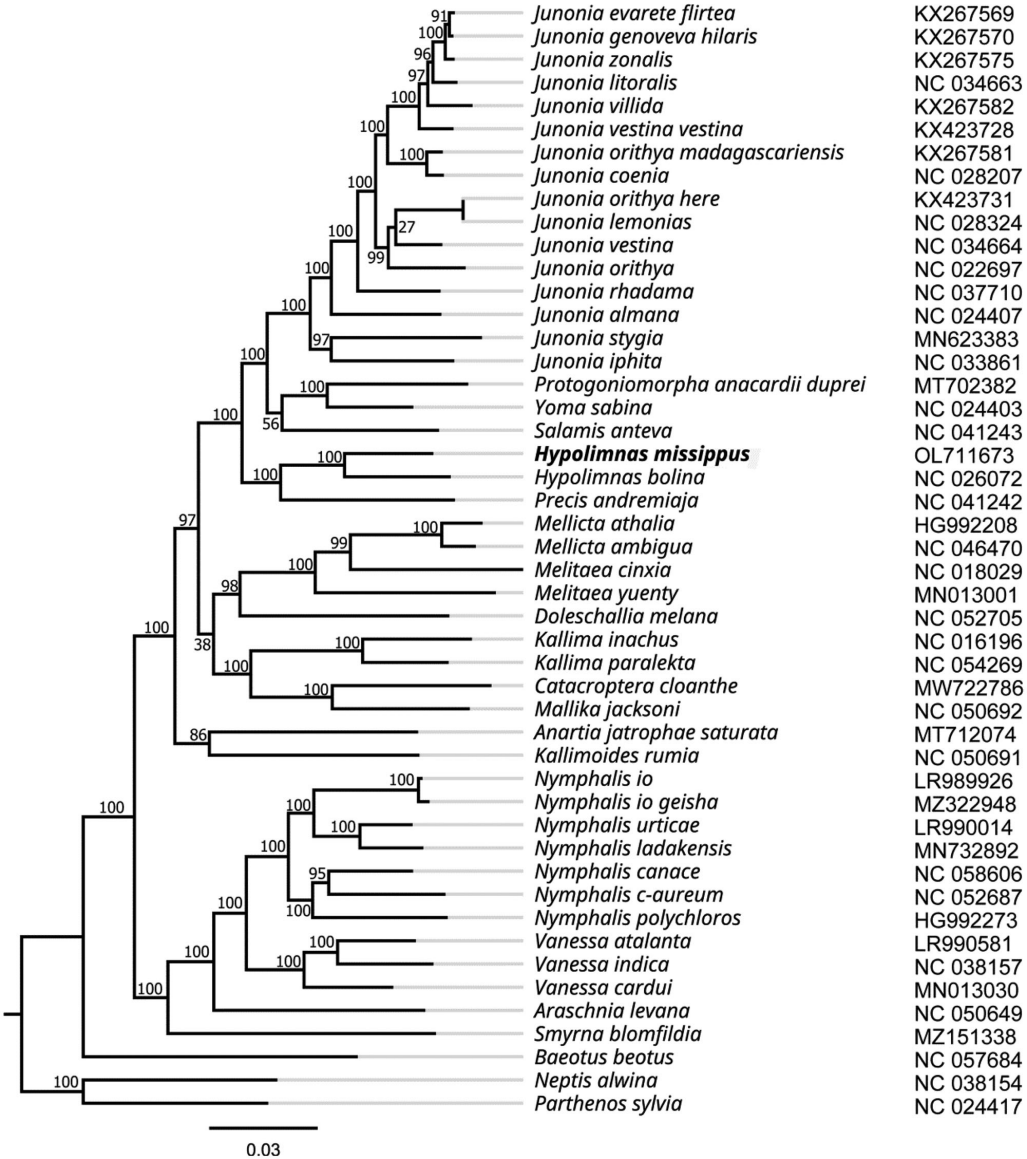
Maximum-likelihood phylogenetic tree based on whole mitogenome from 46 *Nymphalinae* butterfly and two outgroup butterfly (*Neptis alwina* and *Parthenos sylvia*) and the support values are shown at the branches.

### 3.2. Phylogenetic analysis

The phylogenetic position of *H. misippus* was inferred by a ML phylogenetic tree using FastTree based on the whole mitogenome of 46 Nymphalinae butterflies and two outgroup butterflies (*Neptis alwina* and *Parthenos sylvia*) (Wu et al. 2014; Chen et al. 2020; Hamilton et al. 2020; Liu et al. 2020; Li et al. 2020; Alexiuk et al. 2021; Lalonde 2021; Lohse et al. 2021; Aguila et al. 2021). As shown in Figure 2, the ML phylogenetic tree shows that *H. misippus* was most closely related to *H. bolina*, with support values of 100%. Furthermore, the genus *Hypolimnas* were clustered together with *Precis andremiaja*. This relationships is congruent with previous phylogenetic studies (Kim et al. 2021).

## 4. Conclusion

The complete mitochondrial genome of *H. misippus* was sequenced on the BIG-500 platform to generate a 15,283 bp mitogenome (Genbank accession no. OL711673). The phylogenetic position of *H. misippus* within the subfamily of Nymphalinae was determined, and the results showed that *H. misippus* was closely related to *H. bolina*. Our findings are helpful to understand the phylogenetic status of *H. misippus*. It also provides baseline molecular data for future studies on the evolutionary relationships within the Nymphalidae.

## Ethical approval

This study does not involve Endangered or protected species according to IUZN (2021). The approval of sample collection is not required according to the Animal Ethical and Welfare Committee of Southwest Forestry University.

## Supplementary Material

Supplemental Material

## Data Availability

The genome sequence data that support the findings of this study are openly available in GenBank of NCBI (https://www.ncbi.nlm.nih.gov) under accession no. OL711673. The associated BioProject, SRA, and Bio-Sample numbers are PRJNA789424, SRR17245425, and SAMN24109441 respectively.
