# Supplementary figures and images for: Characterization of the complete mitochondrial genome of the Hypolimnas misippus Linnaeus 1764 (Lepidoptera: nymphalidae)
Source: Mitochondrial DNA B Resour. 2023 Aug 24;8(8):895–8. doi: 10.1080/23802359.2023.2246673 (PMC10453977; doi:10.1080/23802359.2023.2246673)

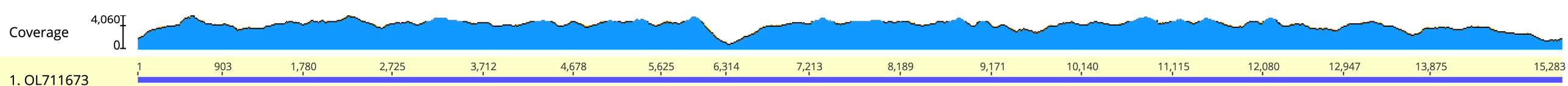

Supplement: Supplemental Material [file TMDN_A_2246673_SM5883.tiff]
